# Ir-6: A Novel Iridium (III) Organometallic Derivative for Inhibition of Human Platelet Activation

**DOI:** 10.1155/2018/8291393

**Published:** 2018-05-02

**Authors:** Ren-Shi Shyu, Themmila Khamrang, Joen-Rong Sheu, Chih-Wei Hsia, Marappan Velusamy, Chih-Hsuan Hsia, Duen-Suey Chou, Chao-Chien Chang

**Affiliations:** ^1^Division of Nephrology, Department of Internal Medicine, Min-Sheng General Hospital, Taoyuan 330, Taiwan; ^2^Graduate Institute of Medical Sciences, College of Medicine, Taipei Medical University, Taipei 110, Taiwan; ^3^Department of Chemistry, North Eastern Hill University, Shillong 793022, India; ^4^Department of Pharmacology, School of Medicine, College of Medicine, Taipei Medical University, Taipei 110, Taiwan; ^5^Department of Cardiology, Cathay General Hospital, Taipei 106, Taiwan

## Abstract

Platelet activation has been reported to play a major role in arterial thrombosis, cancer metastasis, and progression. Recently, we developed a novel Ir(III)-based compound, [Ir(Cp^∗^)1-(2-pyridyl)-3-(4-dimethylaminophenyl)imidazo[1,5-a]pyridine Cl]BF_4_ or Ir-6 and assessed its effectiveness as an antiplatelet drug. Ir-6 exhibited higher potency against human platelet aggregation stimulated by collagen. Ir-6 also inhibited ATP-release, intracellular Ca^2+^ mobilization, P-selectin expression, and the phosphorylation of phospholipase C*γ*2 (PLC*γ*2), protein kinase C (PKC), v-Akt murine thymoma viral oncogene (Akt)/protein kinase B, and mitogen-activated protein kinases (MAPKs), in collagen-activated platelets. Neither the adenylate cyclase inhibitor SQ22536 nor the guanylate cyclase inhibitor 1H-[1,2,4]oxadiazolo[4,3-a]quinoxalin-1-one significantly reversed the Ir-6-mediated inhibition of collagen-induced platelet aggregation. Moreover, Ir-6 did not considerably diminish OH radical signals in collagen-activated platelets or Fenton reaction solution. At 2 mg/kg, Ir-6 markedly prolonged the bleeding time in experimental mice. In conclusion, Ir-6 plays a crucial role by inhibiting platelet activation through the inhibition of signaling pathways, such as the PLC*γ*2–PKC cascade and the subsequent suppression of Akt and MAPK activation, thereby ultimately inhibiting platelet aggregation. Therefore, Ir-6 is a potential therapeutic agent for preventing or treating thromboembolic disorders or disrupting the interplay between platelets and tumor cells, which contributes to tumor cell growth and progression.

## 1. Introduction

Platelets are anucleate blood cells that play a crucial role in thrombosis under both physiological and pathological conditions. They are required for maintaining the integrity of the vascular system and are the first-line of defense against hemorrhage. On encountering a subendothelial matrix exposed by an injury to a blood vessel, platelets adhere to the matrix and become activated and adhesive to other platelets, leading to further aggregation [1]. During platelet activation, the release of several mediators (e.g., ATP and thromboxane A_2_) occurs simultaneously with relative intracellular Ca^2+^ ([Ca^2+^]_i_) mobilization, attracting additional platelets to the site of the injured endothelium and consequently thickening the initial platelet monolayer. Finally, fibrinogen binds to its specific platelet receptor, completing the final common pathway for platelet aggregation.

Platelets and their activation are the key events, which play critical role in cancer progression [2, 3]. The effect of platelets on malignancy development has been proposed to be a controlled process that triggers cancer growth. Therefore, inhibition of platelet aggregation is expected to be a novel therapeutic target for reducing the formation of platelet–tumor complexes [4]. Transition metal complexes, including those of iridium (Ir), have been explored for at least a decade as platforms for producing innovative molecules with anticancer properties. Although several biological studies have shown that Ir-based compounds exhibit potent anticancer activity with relatively few side effects, no study has investigated the effects of Ir compounds on platelet aggregation to date.

**Figure 1:**
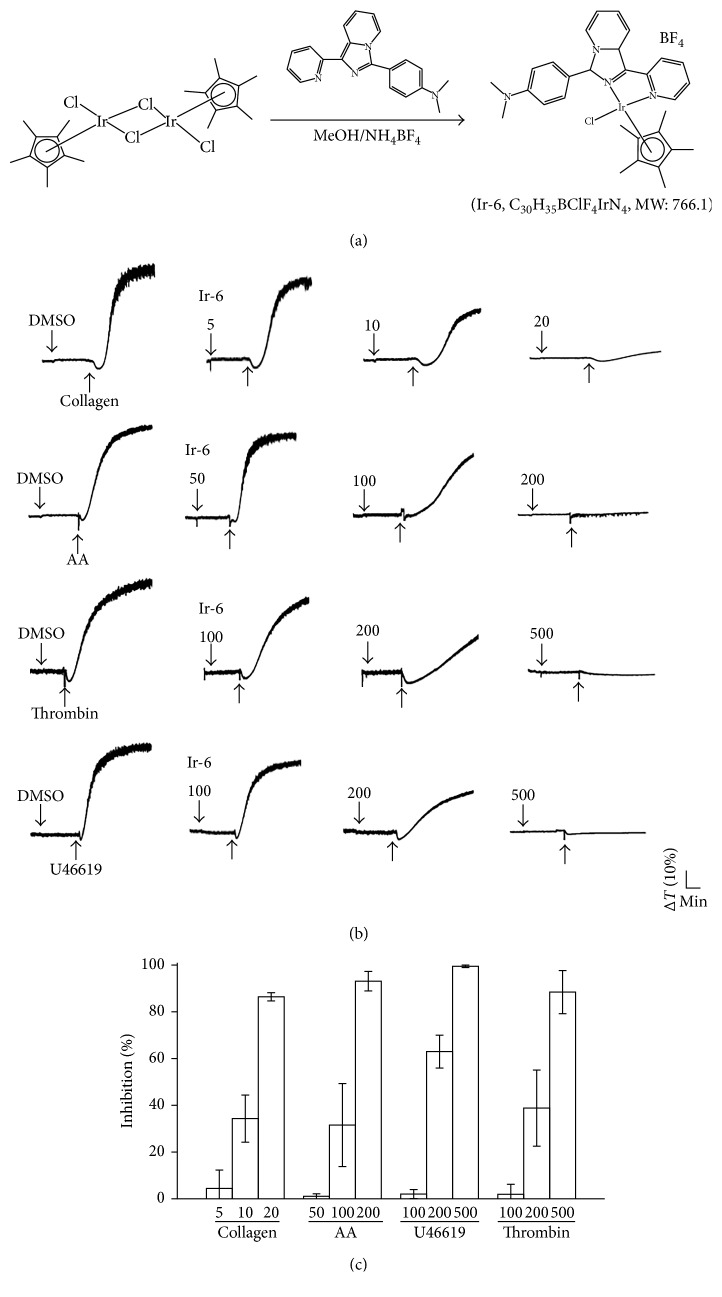
Comparison among the levels of Ir-6-mediated inhibition of platelet aggregation induced by agonists in washed human platelets. (a) Synthesis of complex (Ir-6) [Ir(Cp^∗^)(L)Cl]BF_4_. (b) Platelets (3.6 × 10^8^ cells/mL) were preincubated with the solvent control (0.1% DMSO) or Ir-6 (5–500 *μ*M) and subsequently treated with collagen (1 *μ*g/mL), arachidonic acid (AA; 120 *μ*M), thrombin (0.01 U/mL), or U46619 (1 *μ*M) to stimulate platelet aggregation. (c) Concentration–response histograms of Ir-6 representing the levels of inhibition of platelet aggregation stimulated by various agonists. Data are presented as the mean ± standard the error of the mean (*n*=4).

In recent years, researchers have paid more attention on Ir(III) compounds since they found powerful antitumor activity with low cytotoxicity toward normal tissues [5, 6]. Likewise, Ir complexes show excellent antiangiogenic effects through activating various antiangiogenic signaling pathways [5]. In our earlier studies, we have shown that phenol [7] and anisole [8] substituted imidazo[1,5-a]pyridine ligand-based iridium(III) complexes and their antiplatelet and antithrombotic activities. In continuation of these studies on the effect of electron donors on imidazo[1,5-a]pyridine-based ligand, we introduced dimethyl aniline as strong electron donor group and studied its antiplatelet activities. In addition, we developed a new biologically active Ir(III) derivative Ir-6 as shown in Figure 1(a). Moreover, a previous study had reported the photophysical and photochemical properties of Ir(III)-cyclometalated complexes that contain the luminescent ligands [9]. Hence, we used the same ligand to synthesise the Ir-6 complex [Ir(Cp^∗^)1-(2-pyridyl)-3-(4-dimethylaminophenyl)imidazo[1,5-a] pyridine Cl]BF4 to perform this study. Although several in vitro and in vivo anticancer activities have demonstrated in Ir-based compounds, to date, no study has investigated their effects on platelet aggregation. Our preliminary findings revealed the potent antiplatelet activity of Ir-6 in human platelets; hence, we further examined the molecular mechanism and activity of Ir-6 against platelet activation. The present study is a primary step for investigating whether the potent activity of Ir-6 against cancer progression is due to its ability to inhibit platelet aggregation effectively.

## 2. Materials and Methods

### 2.1. Chemicals

Thrombin, collagen, arachidonic acid (AA), luciferin-luciferase, U46619, phorbol 12,13-dibutyrate (PDBu), nitroglycerin (NTG), heparin, prostaglandin E_1_ (PGE_1_), 5,5-dimethyl-1-pyrroline N-oxide (DMPO), SQ22536, 1H-[1, 2, 4]oxadiazolo[4,3-a]quinoxalin-1-one (ODQ), LY294002, SB203580, PD98059, SP600125, and bovine serum albumin (BSA) were purchased from Sigma (St. Louis, MO, USA). Fura-2AM was purchased from Molecular Probes (Eugene, OR, USA). An anti-phospho-p38 mitogen-activated protein kinase (MAPK) Ser^182^ monoclonal antibody (mAb) was purchased from Santa Cruz Biotechnology (Santa Cruz, CA, USA). Anti-p38 MAPK, anti-phospho-c-Jun N-terminal kinase (JNK) (Thr^183^/Tyr^185^), and anti-p44/42 extracellular signal-regulated kinase (ERK) mAbs as well as anti-phospholipase C*γ*2 (PLC*γ*2), anti-phospho (Tyr^759^) PLC*γ*2, anti-phospho-(Ser) protein kinase C (PKC) substrate (pleckstrin; p-p47), anti-JNK, and anti-phospho-p44/p42 ERK (Thr^202^/Tyr^204^) polyclonal antibodies (pAbs) were purchased from Cell Signaling (Beverly, MA, USA). Anti-phospho-protein kinase B (Akt) (Ser^473^) and anti-Akt mAbs were purchased from Biovision (Mountain View, CA, USA). An anti-pleckstrin (p47) pAb was purchased from GeneTex (Irvine, CA, USA). Hybond-P polyvinylidene fluoride (PVDF) membranes horseradish peroxidase- (HRP-) conjugated donkey anti-rabbit immunoglobulin G (IgG) and sheep anti-mouse IgG were purchased from Amersham (Buckinghamshire, UK). A fluorescein isothiocyanate (FITC) anti-human CD42P (P-selectin) mAb was purchased from BioLegend (San Diego, CA, USA).

### 2.2. Synthesis of [Ir(Cp^∗^)(L)Cl]BF_4_(Ir-6)

To 10 mL of a methanolic solution of 1-(2-pyridyl)-3-(4-dimethylaminophenyl)imidazo[1,5-a]pyridine (L) (0.12 g, 0.4 mM) [9], a solution of [Ir(Cp^∗^)(Cl)_2_]_2_ (0.16 g, 0.2 mM) in 10 mL methanol was added dropwise, and the solution was stirred at room temperature for 3 h. Subsequently, NH_4_BF_4_ (200 mg, 0.6 mM) was added to the solution, and the solution slowly changed color from pale yellow to orange. After 24 h, the solution was evaporated, and the solid obtained was filtered. The residue was washed with diethyl ether (40 mL) and dried under vacuum. The desired products were recrystallized from a mixture of dichloromethane and hexane as orange microcrystals. ^1^H NMR (400 MHz, dimethyl sulfoxide [DMSO]-d6) *δ* 8.86–8.84 (*d*, 1H, *J*=8 Hz), 8.50–8.44 (*m*, 2H), 8.21–8.18 (*t*, 1H, *J*=6 Hz), 8.01–7.99 (*d*, 2H, *J*=8 Hz), 7.59–7.49 (*m*, 2H), 7.19–7.16 (*t*, 1H, *J*=6 Hz), 7.03–7.01 (*d*, 2H, *J*=8 Hz), 3.6 (*s*, 15H), 1.36 (*s*, 6H); UV–Vis (*λ*_abs_, nm) (*ε*, M^−1^·cm^−1^): 400 (1447), 304 (2576), 242 (1468); ESI-MS (m/z): 677.17 [M-BF_4_]^+^ (Figure 1(a)).

### 2.3. Platelet Aggregation

The institutional review board of Taipei Medical University (TMU-JIRB-N201612050), Taiwan, approved this study and conformed to the directives of the Declaration of Helsinki. All human volunteers involved in this study provided informed consent. Human platelet suspensions were prepared as described previously [10]. Human blood samples were collected from adult volunteers who had not taken any drugs or substances that could affect with the tests for at least 14 days before collecting the samples; the collected blood samples were mixed with an acid-citrate-dextrose solution. After centrifugation, the platelet-rich plasma (PRP) was supplemented with 0.5 *μ*M PGE_1_ and 6.4 IU/mL heparin. Tyrode's solution containing 3.5 mg/mL BSA was used to prepare the final suspension of washed human platelets. The final Ca^2+^ concentration in the Tyrode's solution was 1 mM. Platelet aggregation was assessed using a lumiaggregometer (Payton Associates, Scarborough, ON, Canada) as described previously [10]. The platelet suspensions (3.6 × 10^8^ cells/mL) preincubated with various concentrations of Ir-6 or a solvent control (0.1% DMSO) for 3 min before the addition of various agonists (i.e., collagen). The extent of platelet aggregation was calculated and expressed as a percentage relative to the control (without Ir-6) in light transmission units. In the ATP-release assay, a 20 *μ*L of luciferin–luciferase was added 1 min before the addition of agonist; the amount of ATP release was compared with that released by the control platelets.

### 2.4. Measurement of [Ca^2+^]_i_ Mobilization

The [Ca^2+^]_i_ concentration was determined using Fura-2AM as described previously [10]. In brief, citrated whole blood was centrifuged at 120 ×g for 10 min, and the supernatant was collected and incubated with 5 *μ*M Fura-2AM for 1 h. Human platelet suspensions were prepared as described in the previous section. The Fura-2AM-loaded platelets were washed and preincubated with Ir-6 in the presence of 1 mM CaCl_2_ and stimulated by collagen. Fura-2 fluorescence was measured using a spectrofluorometer (Hitachi FL Spectrophotometer F-4500, Tokyo, Japan) at excitation wavelengths of 340 and 380 nm and an emission wavelength of 510 nm.

### 2.5. Detection of Lactate Dehydrogenase (LDH)

To detect the LDH activity, washed human platelets (3.6 × 10^8^ cells/mL) were preincubated with 20–100 *μ*M Ir-6 or the solvent control (0.1% DMSO) for 20 min at 37°C. An aliquot of the supernatant (10 *µ*L) was placed on a Fuji Dri-Chem slide LDH-PIII (Fuji, Tokyo, Japan), and the absorbance was measured at 540 nm by using a UV-Vis spectrophotometer (UV-160; Shimadzu, Japan). A maximal value (MAX) of LDH was recorded in sonicated platelets.

### 2.6. Platelet Surface P-Selectin Expression Analysis by Flow Cytometric

To this analysis, washed platelet suspensions were prepared as described previously [8]. Aliquots of the platelet suspensions (3.6 × 10^8^ cells/mL) were preincubated with Ir-6 (10 and 20 *µ*M) or the solvent control (0.1% DMSO) and FITC-P-selectin (2 *µ*g/mL) for 3 min. Collagen (1 *µ*g/mL) was subsequently added to trigger platelet activation. The suspensions were then assayed for fluorescein-labeled platelets by using a flow cytometer (FAC Scan System, Becton Dickinson, San Jose, CA, USA). Data were collected from 50,000 platelets per experimental group, and the platelets were distinguished according to their characteristic forward and orthogonal light-scattering profiles. All experiments were repeated at least four times to ensure reproducibility.

### 2.7. Immunoblotting

For the immunoblotting analysis, washed platelets (1.2 × 10^9^ cells/mL) were preincubated with Ir-6 (10 and 20 *µ*M) or the solvent control (0.1% DMSO) for 3 min, and then collagen was added to trigger platelet activation. After 10 min, the reaction was stopped by adding EDTA, and the resulting platelets were resuspended in 200 *μ*L lysis buffer. Proteins (approximately 80 *μ*g) were separated through sodium dodecyl sulfate polyacrylamide gel electrophoresis (SDS-PAGE) on a 12% gel. The separated proteins were transferred to PVDF membranes by using a Bio-Rad semidry transfer unit (Bio-Rad, Hercules, CA, USA). The membranes were then blocked with Tris-buffered saline in Tween 20 (TBST; 10 mM Tris-base, 100 mM NaCl, and 0.01% Tween 20) containing 5% BSA for 1 h and were then probed using various primary antibodies. The membranes were subsequently incubated with HRP-conjugated anti-mouse IgG or anti-rabbit IgG (diluted 1 : 3000 in TBST) for 1 h. An enhanced chemiluminescence system was used to detect immunoreactive bands, and the optical densities (OD) of the bands were quantified using Bio-profil Biolight (version V2000.01; Vilber Lourmat, Marne-la-Vallée, France).

### 2.8. Detection of OH· Radical Formation in Both Platelets and Fenton Reaction Solution by Electron Spin Resonance Spectrometry

Electron spin resonance (ESR) spectrometry (Bruker EMX ESR, Billerica, MA, USA) analysis was performed as described previously [11]. Either platelet suspension (3.6 × 10^8^ cells/mL) or Fenton reaction solution (50 *μ*M FeSO_4_ + 2 mM H_2_O_2_) was preincubated with 0.1% DMSO or Ir-6 (10 and 20 *μ*M) for 3 min, with or without the addition of 1 *μ*g/mL collagen. After 5 min, 100 *μ*M DMPO was added to the suspensions before ESR spectrometry. The ESR spectral signals were recorded using a quartz flat cell designed for aqueous solutions. The spectrometer was operated at 20 mW and 9.78 GHz, with a scan range of 100 G and a receiver gain of 5 × 10^4^. The modulation amplitude was 1 G, and the time constant was 164 ms. Each sample was scanned for 42 s, and each spectrum was the sum of three scans.

### 2.9. Tail Bleeding Time Assay in Mice

To investigate whether Ir-6 has bleeding risk, the bleeding time assay was performed through transection of the tails in male ICR mice. To this, after 30 min of intraperitoneal administration of Ir-6 (1 or 2 mg/kg), the tails of mice were cut at a 3 mm distance from the tip. The tails were directly placed in tubes filled with normal saline at 37°C to measure the bleeding time, which was recorded until the bleeding completely stopped. The animal experiments were conformed to the Guide for the Care and Use of Laboratory Animals (8th edition, 2011) and obtained an affidavit of approval for animal use from Taipei Medical University (LAC-2016-0395), Taiwan.

### 2.10. Statistical Analysis

The experimental results are expressed as the mean ± standard error of the means (S.E.M.) and are accompanied by the number of observations (*n*). The values of *n* refer to the number of experiments, and each experiment was conducted using different blood donors. The unpaired Student's *t*-test was used to determine the significance of differences between the control and experimental mice. The differences between multiple groups in other experiments were assessed through analysis of variance (ANOVA). When ANOVA indicated significant differences among the group means, the groups were compared using the Student–Newman–Keuls method. In the analysis, *P* values < 0.05 were considered statistically significant. Statistical analyses were performed using SAS (version 9.2; SAS Inc., Cary, NC, USA).

## 3. Results

### 3.1. Effects of Ir-6 on Platelet Aggregation in Washed Human Platelets

As shown in Figures 1(b) and 1(c), pretreatment of Ir-6 (5–20 *μ*M) intensely and concentration-dependently inhibited collagen-induced aggregation in washed human platelets. In response to 120 *µ*Μ AA stimulation, Ir-6 gradually inhibited aggregation of platelets even at 50–200 *μ*M. Furthermore, at 100–500 *μ*M, Ir-6 exhibited relatively weak activity against platelet aggregation induced by 0.01 U/mL thrombin or 1 *µ*Μ U46619, a prostaglandin endoperoxide analogue; this result indicated that Ir-6 had more potent activity against collagen-induced aggregation than *t* that induced by other agonists AA, thrombin, and U46619 (Figure 1(c)). The used solvent control (0.1% DMSO) did not ominously affect platelet aggregation. In the subsequent experiments, 1 *μ*g/mL collagen was used as an agonist for investigating the possible mechanisms through which Ir-6 inhibits human platelet activation.

### 3.2. Ir-6 Inhibits ATP Release, Relative [Ca^2+^]_i_ Mobilization, and Surface P-Selectin Expression

**Figure 2:**
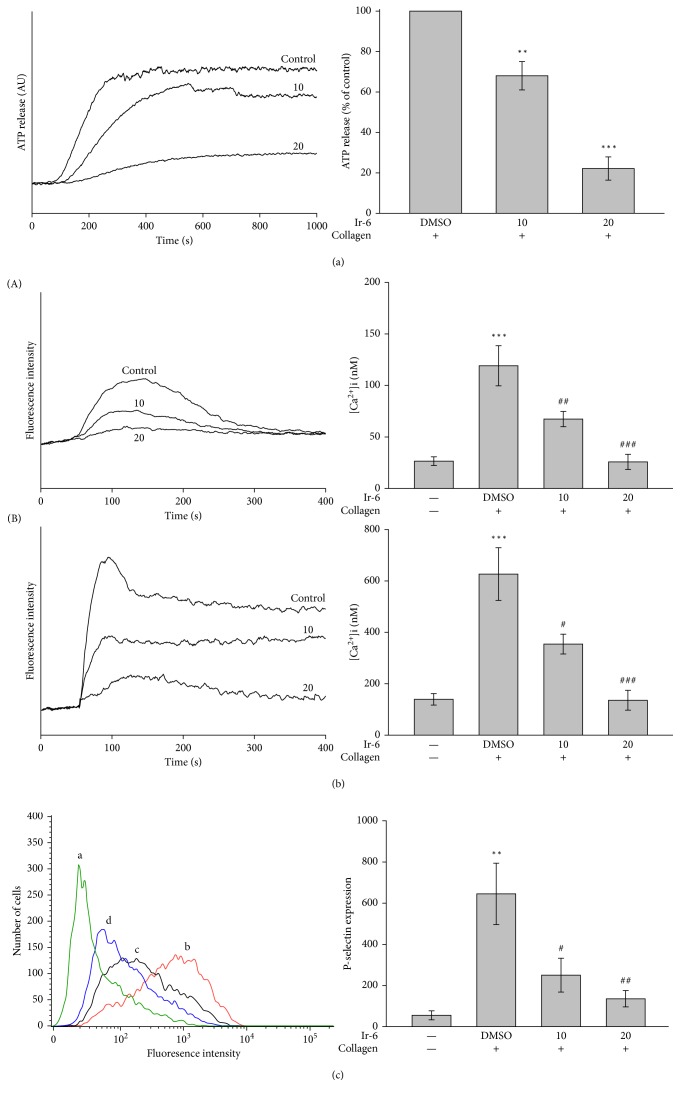
Effect of Ir-6 on ATP-release reaction, relative [Ca^2+^]_i_ mobilization, and surface FITC-P-selectin expression in washed human platelets. Platelets (3.6 × 10^8^ cells/mL) were preincubated with the solvent control (0.1% DMSO) or Ir-6 (10 and 20 *µ*M). Subsequently, collagen (1 *μ*g/mL) was added to trigger either (a) the ATP-release reaction (AU; arbitrary unit) or (b) relative [Ca^2+^]_i_ mobilization (nM) in calcium-free Tyrode's solution (A) or Tyrode's solution (B). (c) Washed platelets (3.6 × 10^8^/mL) were preincubated with the solvent control (0.1% DMSO) or Ir-6 (10 and 20 *µ*M) and FITC-P-selectin (2 *µ*g/mL) for 3 min and then stimulated using collagen (1 *μ*g/mL). The corresponding statistical data are shown in the right panel of each Figures 2(a)–2(c). Data are presented as the mean ± standard error of the mean (*n*=4). ^∗^*P* < 0.01 and ^∗∗∗^*P* < 0.001, compared with the DMSO-treated group (a) or resting control (b and c); ^#^*P* < 0.05, ^##^*P* < 0.01, and ^###^*P* < 0.001, compared with the DMSO-treated group (b and c).

Platelet activation is connected with the release of granular contents such as ATP and Ca^2+^ and surface P-selectin expression, which leads to strong platelet aggregation. In the present study, Ir-6 (10 and 20 *µ*M) inhibited the ATP-release reaction stimulated by 1 *μ*g/mL collagen (Figure 2(a)). Collagen stimulates [Ca^2+^]_i_ by promoting the entry of Ca^2+^ into the cytosol from two resources; Ca^2+^ is released from intracellular stores and enters to the platelet across the cell membrane.  Figure 2(b), (A) shows responses to collagen without 1 mM CaCl_2_. Under this condition, influx of calcium is expected to be markedly diminished, and therefore any change in [Ca^2+^]_i_ may be attributed to discharge from intracellular stores into the cytoplasma. Pretreatment with IL-6 (10 and 20 *μ*M) obviously reduced relative [Ca^2+^]_i_ mobilization both in calcium-free Tyrode's solution (resting control, 26.5 ± 4.3 nM; collagen-stimulated, 119.1 ± 19.5 nM; 10 *μ*M Ir-6, 67.3 ± 7.3 nM; and 20 *μ*M Ir-6, 25.8 ± 7.4 nM; *n*=4), Figure 2(b), (A) and in Tyrode's solution (resting control, 139.4 ± 22.4 nM; collagen-stimulated, 626.8 ± 102.6 nM; 10 *μ*M Ir-6, 354.3 ± 38.7 nM; and 20 *μ*M Ir-6, 135.6 ± 38.7 nM; *n*=4), Figure 2(b), (B) in platelets stimulated by 1 *μ*g/mL collagen. In addition, in quiescent (resting) platelets, P-selectin is located on the inner wall of the *α*-granules. Platelet activation exposes the inner walls of the granules to the outside of the cell [12]. Ir-6 treatment markedly reduced collagen-induced surface P-selectin expression, as demonstrated by the statistical data in the right panel of Figure 2(c) (resting control, 55.7 ± 21.7; collagen-activated, 645.0 ± 148.9; 10 *μ*M Ir-6, 250.7 ± 82.0; 20 *μ*M Ir-6, 136.0 ± 39.7; *n*=4).

### 3.3. Effect of Ir-6 on Cyclic Nucleotide Formation and LDH Release in Washed Human Platelets

**Figure 3:**
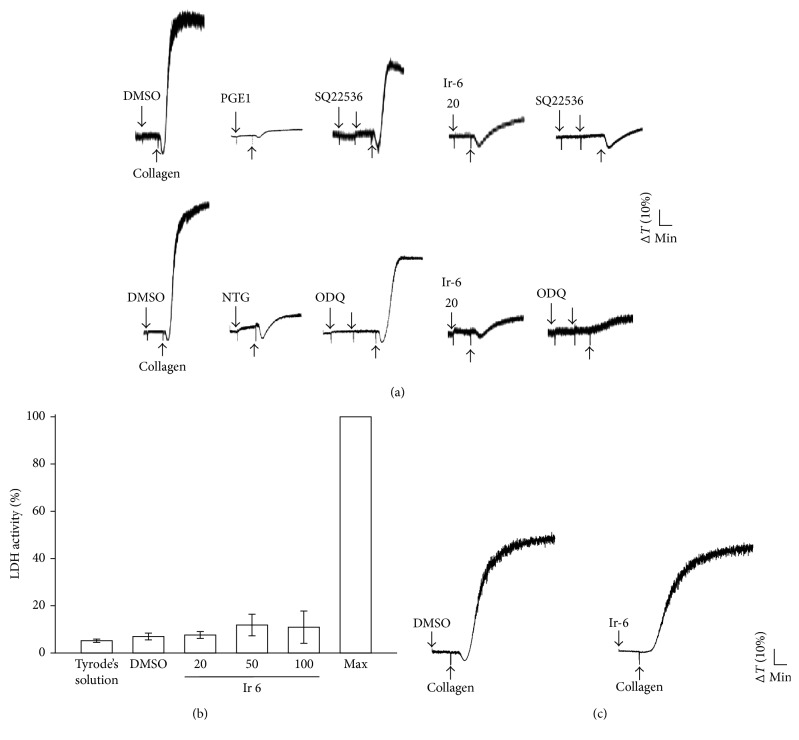
Effect of Ir-6 on cyclic nucleotide formation, LDH release, and cytotoxicity in human platelets. (a) Washed platelets (3.6 × 10^8^ cells/mL) were preincubated with 1 *μ*M PGE1, 10 *μ*M NTG, or 20 *µ*M Ir-6 with or without 100 *μ*M SQ22536 or 10 *μ*M ODQ and were subsequently treated with collagen (1 *μ*g/mL) to induce platelet aggregation. (b) Washed platelets (3.6 × 10^8^ cells/mL) were preincubated with the solvent control (0.1% DMSO) or with various concentrations of Ir-6 (20, 50, and 100 *µ*M) for 20 min, and a 10 *µ*L aliquot of the supernatant was deposited on a Fuji Dri-Chem slide LDH-PIII. (c) For other experiment, washed platelets were preincubated with the solvent control (0.1% DMSO) or Ir-6 (100 *μ*M) for 10 min and subsequently washed two times with Tyrode's solution. Collagen (1 *μ*g/mL) was then added to trigger platelet aggregation. Data are presented as the mean ± standard error of the mean (*n*=4). Profiles in (a) and (c) represent four independent experiments.

Both 100 *μ*M SQ22536, an adenylate cyclase inhibitor and 10 *μ*M ODQ, a guanylate cyclase inhibitor, significantly reversed the inhibition of collagen-induced platelet aggregation mediated by 1 *μ*M PGE_1_ or 10 *μ*M NTG (Figure 3(a)). However, neither SQ22536 nor ODQ significantly reversed the inhibition of collagen-induced platelet aggregation mediated by 20 *μ*M Ir-6 (Figure 3(a)), indicating that the mechanisms of Ir-6-mediated inhibition of platelet aggregation do not involve the enhancement of cyclic nucleotide synthesis. Moreover, the aggregation curves of platelets preincubated with 100 *μ*M Ir-6 for 10 min and subsequently washed two times with Tyrode's solution were not significantly different from those of platelets preincubated with the solvent control (0.1% DMSO) under equivalent conditions (Figure 3(c)). This finding indicates that the effects of Ir-6 on platelet aggregation are reversible and noncytotoxic. Furthermore, the LDH release results revealed that incubating platelets for 20 min with Ir-6 (20, 50, and 100 *μ*M) did not significantly increase LDH activity or exhibit cytotoxic effects on the platelets (Figure 3(b)), demonstrating that Ir-6 does not affect platelet permeability or induce platelet cytolysis.

### 3.4. Activity of Ir-6 on Regulating PLC*γ*2–PKC Signaling and Akt Activation

**Figure 4:**
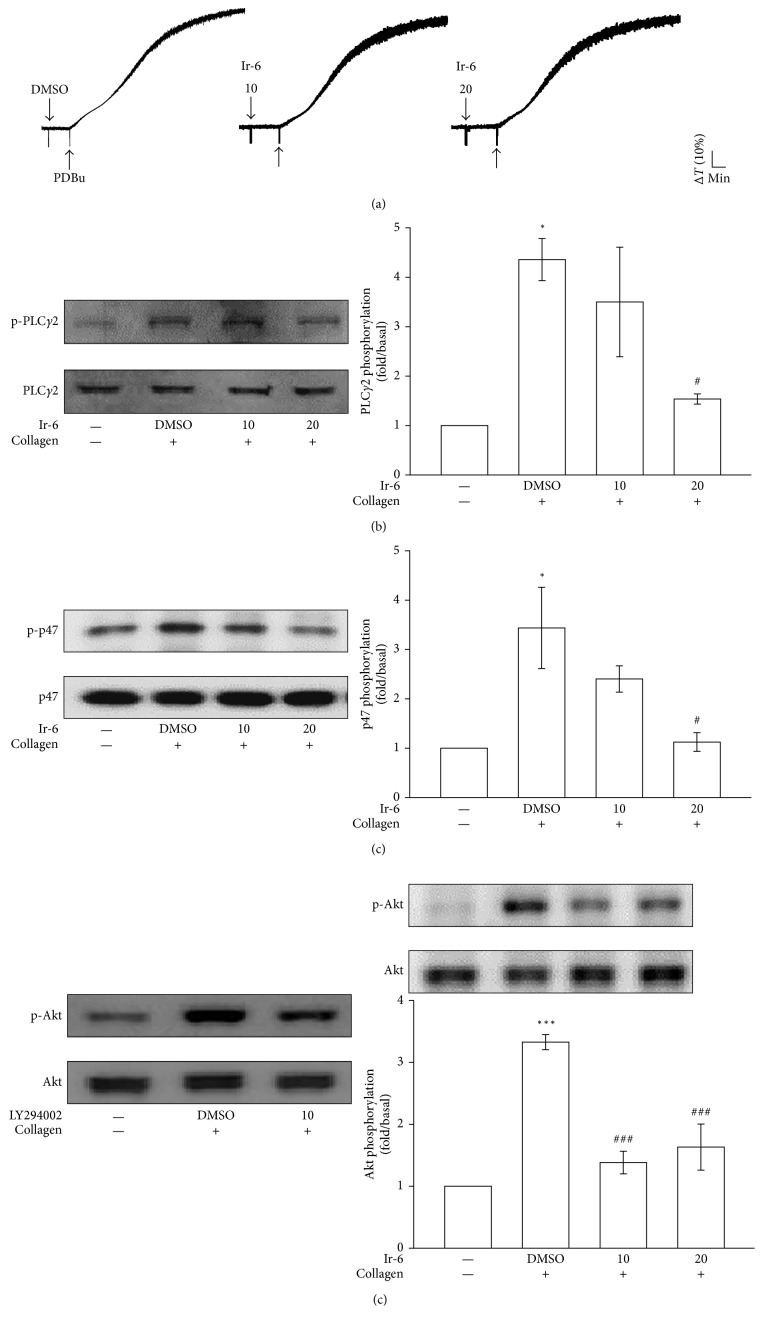
Effects of Ir-6 on PLC*γ*2, PKC, and Akt activation in human platelets. Washed platelets were preincubated with the solvent control (0.1% DMSO), LY294002 (10 *µ*M), or Ir-6 (10 or 20 *µ*M). Subsequently, either collagen (1 *μ*g/mL) or PDBu (150 nM) was added to trigger (a) platelet aggregation, (b) PLC*γ*2 phosphorylation, and (c) PKC activation (pleckstrin phosphorylation), or (d) Akt activation. Platelets were collected, and their subcellular extracts were analyzed to determine the levels of phosphorylation of (b) PLC*γ*2, (c) PKC, and (d) Akt. Data are presented as the mean ± standard error of the mean (*n*=4). ^∗^*P* < 0.05 and ^∗∗∗^*P* < 0.001, compared with the resting control; ^#^*P* < 0.05 and ^###^*P* < 0.001, compared with the DMSO-treated group. The profiles in (a) and (d, left panel) are representative of four independent experiments.

PLCs hydrolyze phosphatidylinositol 4,5-bisphosphate to generate the secondary messengers inositol 1,4,5-trisphosphate (IP_3_) and diacylglycerol (DAG). IP_3_ triggers relative [Ca^2+^]_i_ mobilization, while DAG activates PKC. PKC activation yields a protein, approximately 47 kDa in size, that is predominantly phosphorylated (p47 protein; pleckstrin) and causes the ATP-release reaction [13]. As shown in Figure 4(a), treatment of platelets with 10 or 20 *µ*M Ir-6 did not significantly inhibit aggregation induced by 150 nM PDBu, a PKC activator (Figure 4(a)), indicating that Ir-6 does not directly disrupt PKC activation. Figures 3(a) and 3(b) illustrates the inhibitory effects of Ir-6 on ATP release and relative [Ca^2+^]_i_ mobilization induced by collagen. Therefore, we further investigated the effect of Ir-6 on the phosphorylation of the PLC*γ*2–PKC signaling cascade. At 10 and 20 *µ*M, Ir-6 markedly reduced PLC*γ*2 phosphorylation as well as PKC activation (pleckstrin phosphorylation) in collagen-stimulated platelets (Figures 4(b) and 4(c)). Akt is a serine/threonine-specific protein kinase that plays a key role in various cellular processes, such as platelet activation, cell proliferation, apoptosis, and cell migration [14]. Both LY294002 (an inhibitor of Akt; 10 *µ*M) and Ir-6 (10 and 20 *µ*M) markedly inhibited collagen-induced Akt phosphorylation (Figure 4(d)), demonstrating the crucial role of inhibition of the Akt signaling pathway in the Ir-6-mediated inhibition of platelet activation.

### 3.5. Effects of Ir-6 on Inhibiting p38 MAPK, ERK2, and JNK1 Phosphorylation

**Figure 5:**
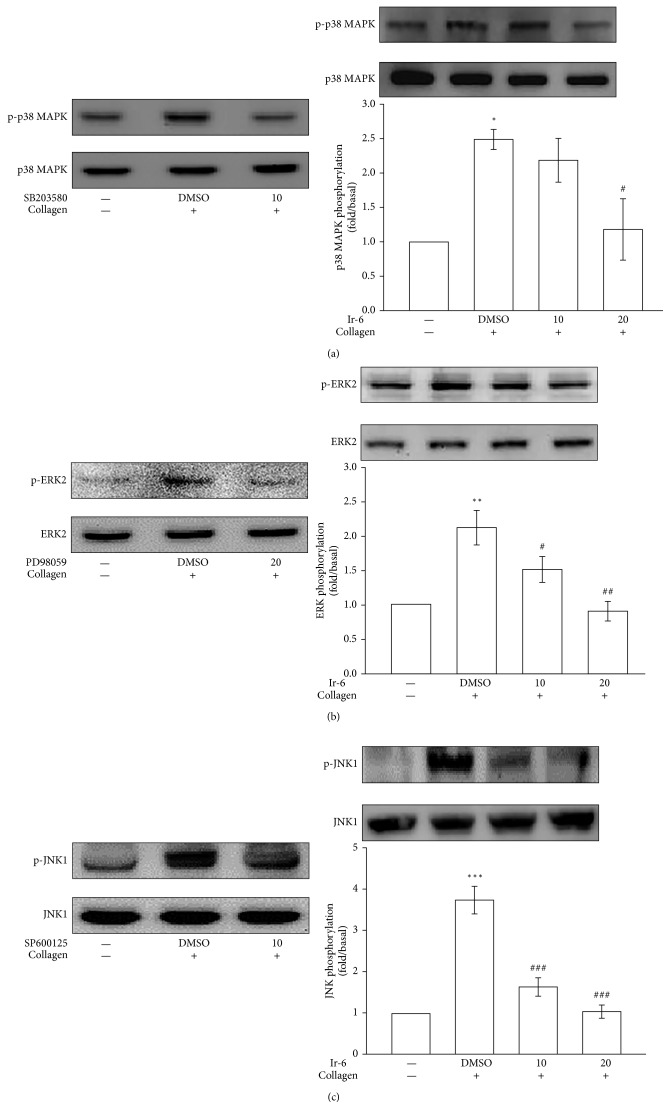
Inhibitory effects of Ir-6 on the phosphorylation of p38 MAPK, ERK2, and JNK1 in human platelets. The washed platelets (1.2 × 10^9^ cells/mL) were preincubated with the solvent control (0.1% DMSO), SB203580 (10 *µ*M), PD98059 (20 *µ*M), SP600125 (10 *µ*M), or Ir-6 (10 or 20 *µ*M); subsequently, collagen (1 *μ*g/mL) was added to trigger platelet activation. The platelets were collected, and their subcellular extracts were then analyzed to determine the levels of phosphorylation of (a) p38 MAPK, (b) ERK2, and (c) JNK1. Data are presented as the mean ± standard error of the mean (*n*=4). ^∗^*P* < 0.05, ^∗∗^*P* < 0.01, and ^∗∗∗^*P* < 0.001, compared with the resting control; ^#^*P* < 0.05, ^##^*P* < 0.01, and ^###^*P* < 0.001, compared with the DMSO-treated group. The profiles of left panels are representative of four independent experiments.

Several signaling molecules of the MAPK phosphorylation pathway were evaluated to investigate the inhibitory mechanisms of Ir-6 in platelet activation. In eukaryotic organisms, MAPKs (p38 MAPK, ERKs, and JNKs) control major cellular reactions and contribute to various events of cell proliferation, migration, differentiation, and apoptosis. ERKs, JNK1, and p38 MAPK have been identified in platelets [15]. SB203580 (an inhibitor of p38 MAPK; 10 *µ*M), PD98059 (an inhibitor of ERK2; 20 *µ*M), and SP600125 (an inhibitor of JNK1; 10 *µ*M) markedly inhibited p38 MAPK (Figure 5(a)), ERK2 (Figure 5(b)), and JNK1 (Figure 5(c)) phosphorylation in collagen-activated platelets, respectively. Ir-6 reduced the phosphorylation of these three proteins in a concentration-dependent manner. Nevertheless, at 10 *µ*M, Ir-6 nonsignificantly inhibited p38 MAPK phosphorylation (Figure 5).

### 3.6. Role of OH· Radical on Ir-6-Mediated Inhibition of Platelet Aggregation

**Figure 6:**
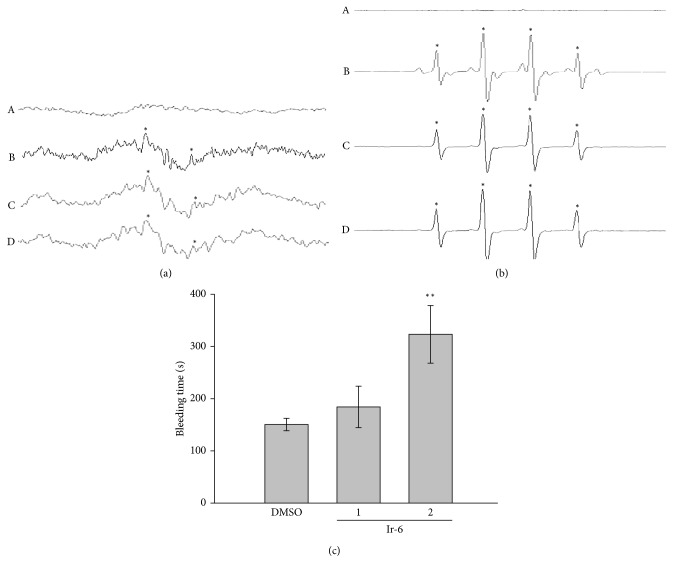
Regulatory activities of Ir-6 on OH· formation in either platelet suspensions or Fenton reaction solution and determination of bleeding time in experimental mice. (a) Washed platelets or (b) the Fenton reaction solution was preincubated with (A) Tyrode's solution (resting control), (B) 0.1% DMSO, (C) 10 *µ*M Ir-6, or (D) 20 *µ*M Ir-6. Collagen (1 *µ*g/mL) was then added into platelet suspensions for the ESR experiments as described in Materials and Methods. Profiles in (a) and (b) are representative of four independent experiments, and an asterisk (^∗^) indicates OH· formation. (c) Mouse tails were transected to measure the bleeding time after 30 min of intraperitoneal administration of either 0.1% DMSO, 1.0 mg/kg Ir-6, or 2.0 mg/kg Ir-6 (all in 50 *μ*L). Data are presented as the mean ± standard error of the mean. *n*=8. ^∗∗^*P* < 0.01, compared with the DMSO (0.1%)-treated group.

An ESR signal *e* of OH· radical formation was observed in both collagen-stimulated platelet suspensions and Fenton reaction solution (cell-free system; Figures 6(a) and 6(b)). A typical OH· signal (*a*^N^ = *a*^H^ = 14.8 G) and a long-lived (*g*=2.005) radical detectable by using DMPO, a spin trap, were observed in collagen-stimulated platelets but not detected in resting platelets (Figure 6(a)), curve (A). Treatment with 10 or 20 *μ*M Ir-6 did not considerably diminish the OH signals in both the platelet suspensions activated by collagen and Fenton reaction solution (Figures 6(a) and 6(b)), suggesting that the Ir-6-mediated inhibition of platelet activation may not be regulated by free radical formation.

### 3.7. Effect of Ir-6 on Bleeding Time

In the tail transection model of mice, after 30 min, the bleeding times were markedly prolonged in mice treated with intraperitoneal administration of 2.0 mg/kg Ir-6 (323.3 ± 55.2 s; *n*=8), but not in those treated with 1.0 mg/kg (184.3 ± 39.7 s; *n*=8) in comparison with mice treated with the solvent control (0.1% DMSO-treated group, 150.5 ± 11.9 s; *n*=8) (Figure 6(c)). Each mouse was monitored whether there was any rebleeding 10 min after the original bleeding stopped.

## 4. Discussion

Platelets activation contributes a major role on thrombotic events among patients with cancer [16]. Chemotherapeutics approach may increase this effect and stimulate vascular thromboembolic events (VTEs) by inducing platelet aggregation, aggravating endothelial damage, and causing vascular toxicity [17]. Of platinum- (Pt-) based chemotherapeutic agents, cisplatin is widely used to a high incidence of treatment-related VTEs [18]. A combination of gemcitabine and Pt-based therapy increased thrombotic and vascular side effects [19, 20]. Thus, research is currently focusing on the development of new metal-based drugs for the inhibition of platelet activation for treating vascular disease, reducing toxic side effects, and overcoming Pt resistance. Notably, this study demonstrated that in addition to its antitumor activity, Ir-6, an Ir(III) derivative, exhibits potent antiplatelet activity.

Platelets adhere to subendothelial matrix proteins (e.g., collagen) which alter platelet shape and cause the release of their granular contents. Collagen mobilizes [Ca^2+^]_i_ to phosphorylate the Ca^2+^/calmodulin-dependent myosin light chain (20 kDa), which is involved in the secretion of granular contents, such as serotonin and ATP [21], and activate platelet aggregation. Therefore, the degree of inhibition of either [Ca^2+^]_i_ mobilization or ATP production is crucial for evaluating the potency of the antiplatelet activity of a compound. In the present study, Ir-6 inhibited platelet aggregation to different degrees, depending on the agonist used to induce aggregation (collagen, U46619, AA, and thrombin), indicating that Ir-6 did not act on the specific individual receptors of these agonists. Therefore, Ir-6 may exert its activity on activated platelets through one or more common signaling pathways.

Platelet activation by collagen substantially alters PLC activation. PLC stimulation results in the production of IP_3_ and DAG. Subsequently, DAG activates PKC and consequently induces p47 phosphorylation [13]. PKC activation triggers specific responses simplifying the transmission of particular upstream signals in individual cellular compartments. The PLC*γ* family comprises the isozymes PLC*γ*1 and PLC*γ*2; PLC*γ*2 is involved in collagen-dependent signaling in platelets [22]. Ir-6 evidently diminished collagen-induced PLC*γ*2–PKC activation; however, Ir-6 did not exert direct effects on PKC activation because it did not interfere with PDBu-induced platelet aggregation. This finding suggests that the Ir-6-mediated inhibition of platelet activation involves PLC*γ*2 downstream signaling. This result may also explain why Ir-6 exhibited higher efficacy in inhibiting platelet activation induced by collagen than in inhibiting that induced by other agonists.

Human platelet activation inhibits through intracellular pathways mediated by cyclic AMP (cAMP) and cyclic GMP (cGMP), and hence these nucleotides are considered to be crucial modulators of platelet activation [23]. Cyclic nucleotides inhibit most of the platelet responses and reduce [Ca^2+^]_i_ levels by enhancing Ca^2+^ uptake, thereby suppressing PLC and PKC activation [23]. Therefore, cAMP and cGMP synergistically inhibit platelet activation. Moreover, neither SQ22536 nor ODQ significantly reversed the Ir-6-mediated inhibition of collagen-induced platelet aggregation. Therefore, Ir-6-mediated mechanisms do not involve the enhancement of cyclic nucleotide synthesis in platelets.

Akt, a downstream effector of phosphoinositide 3-kinase (PI3K) has been reported to show defects in agonist-induced platelet activation when it is deleted in mice, which advocates that Akt normalizes platelet activation; such regulation potentially has consequences in thrombosis [14, 24]. Therefore, specific inhibitors of the Akt isoforms, such as individual PI3K isoforms, may be attractive antithrombotic therapy targets [14]. Cytosolic phospholipase A_2_ (cPLA_2_) is a substrate of p38 MAPK activity induced by agonists such as von Willebrand factor (vWF) and thrombin [25]. Consequently, p38 MAPK is critical for cPLA_2_ stimulation as well as AA release [26]. This observation may explain why Ir-6 had weaker activity in inhibiting p38 MAPK activation as well as thrombin- or AA-stimulated platelet aggregation. Activation of ERK is also an important event involved in platelet aggregation necessitating prior ATP release, which activates P_2_X_1_-mediated Ca^2+^ influx, thereby enhancing the phosphorylation of myosin light-chain kinase [25]. JNK1, another most recently identified MAPK in platelets; therefore, its activation and role are poorly understood. Several agonists such as thrombin, vWF, collagen, and ADP [23] activate JNK1. In addition, a previous study confirmed an increased bleeding time, decreased integrin *α*_IIb_*β*_3_ activation, and severe granule secretion impairment in JNK^−/−^ platelets [27]. Therefore, the inhibition of JNK phosphorylation may play a crucial role in platelet activation. Consistent with these outcomes, the present results demonstrated that Ir-6 markedly inhibits collagen-induced JNK1 phosphorylation.

Reactive oxygen species (hydrogen peroxide) and free radical species (i.e., OH·) act as secondary signals that are involved in platelet activation [28]. In the current study, our ESR spectrometry investigations provided direct evidence demonstrating that Ir-6 does not significantly affect OH· formation in both activated platelets and Fenton reaction solution. Prolongation of hemostatic platelet plug formation (bleeding time) was observed in Ir-6-treated experimental mice. The observed results of bleeding times suggested that the prolongation of bleeding time in humans does not predict the risk of hemorrhage or surgical bleeding. These results question the rationale behind the use of bleeding time for the clinical evaluation of antiplatelet compounds [29].

## 5. Conclusion

In conclusion, the present findings reveal that Ir-6, a novel iridium (III) compound, inhibits platelet activation by preventing signaling molecules, such as the PLC*γ*2–PKC, and subsequently suppresses Akt and JNK1 activation. These alterations inhibit the processes associated with ATP release, [Ca^2+^]_i_ mobilization, and surface P-selectin expression and ultimately inhibit platelet aggregation. However, additional studies are required to investigate other unidentified mechanisms involved in the Ir-6-mediated inhibition of platelet activation. Nevertheless, Ir-6 can be used as a chemotherapeutic agent in cancer treatment. In addition, it can be used as an antiplatelet agent for treating thromboembolic disorders or disrupting the interplay between platelets and tumor cells that contributes to tumor cell growth and progression.
